# Associated Factors of Spontaneous Hemorrhage in Brain Metastases in Patients with Lung Adenocarcinoma

**DOI:** 10.3390/cancers15030619

**Published:** 2023-01-19

**Authors:** Song Soo Kim, Seoyoung Lee, Mina Park, Bio Joo, Sang Hyun Suh, Sung Jun Ahn

**Affiliations:** 1 Department of Radiology, Gangnam Severance Hospital, College of Medicine, Yonsei University, 211 Eonju-ro, Gangnamgu, Seoul 06273, Republic of Korea; 2 Division of Medical Oncology, Department of Internal Medicine, Gangnam Severance Hospital, College of Medicine, Yonsei University, 211 Eonju-ro, Gangnamgu, Seoul 06273, Republic of Korea

**Keywords:** lung cancer, brain metastasis, adenocarcinoma, hemorrhage

## Abstract

**Simple Summary:**

Risk factors of hemorrhage in brain metastases from lung adenocarcinoma has been unknown and how hemorrhage in brain metastases affects patients’ prognosis has not been clarified. We performed a retrospective analysis on 159 BMs and found that hemorrhage in BMs from lung adenocarcinomas may be associated with BM tumor size and a combination of TKI and intracranial radiotherapy. However, BM hemorrhage did not affect OSBM.

**Abstract:**

Background: Hemorrhage in brain metastases (BMs) from lung cancer is common and associated with a poor prognosis. Research on associated factors of spontaneous hemorrhage in patients with BMs is limited. This study aimed to investigate the predictive risk factors for BM hemorrhage and assess whether hemorrhage affects patient survival. Methods: We retrospectively evaluated 159 BMs from 80 patients with lung adenocarcinoma from January 2017 to May 2022. Patients were classified into hemorrhagic and non-hemorrhagic groups. Patient demographics, lung cancer molecular subtype, treatment type, and tumor–node–metastasis stage were compared between the groups. Multivariate generalized estimating equation (GEE) analysis and gradient boosting were performed. To determine whether BM hemorrhage can stratify overall survival after BM (OSBM), univariate survival analysis was performed. Results: In the univariate analysis, hemorrhagic BMs were significantly larger and had a history of receiving combination therapy with tyrosine kinase inhibitor (TKI) and intracranial radiation (*p* < 0.05). Multivariate GEE showed that tumor size and combination therapy were independent risk factors for BM hemorrhage (*p* < 0.05). Gradient boosting demonstrated that the strongest predictor of BM hemorrhage was tumor size (variable importance: 49.83), followed by age (16.65) and TKI combined with intracranial radiation (13.81). There was no significant difference in OSBM between the two groups (*p* = 0.33). Conclusions: Hemorrhage in BMs from lung adenocarcinomas may be associated with BM tumor size and a combination of TKI and intracranial radiotherapy. BM hemorrhage did not affect OSBM.

## 1. Introduction

Brain metastases (BMs) are the most common intracranial tumors in adult patients and typically arise from lung cancer [1,2,3]. Non-small cell lung cancer (NSCLC) constitutes approximately 85% of all lung cancer cases [4]. The prevalence of BMs at initial presentation is 15–20%, and up to 40% of patients eventually develop BMs during the course of NSCLC [5,6]. In particular, patients with adenocarcinoma have a higher risk of BM development than those with other histologic subtypes [7]. Although advanced therapies have been developed that have substantially improved the survival of patients with NSCLC [8], BMs remain an important cause of lung cancer morbidities and are associated with progressive neurologic deficits [9]. BM hemorrhage in particular may lead to serious neurologic deterioration [10] and has been frequently reported in cases of thyroid cancer, melanoma, and renal cell carcinoma [11,12,13]. We anecdotally noted that hemorrhage was a common finding in lung cancer BMs, but its associated factors and clinical significance have not been meticulously investigated.

Hypertension and antithrombogenic medications are known to be significant risk factors for intracranial hemorrhage [14,15]. If these factors lead to BM hemorrhage, hypertension should be controlled and antithrombogenic agents should be avoided. Moreover, with the use of next-generation TKIs, with improved central nervous system penetration, immune check point inhibitors, and sophisticated radiation therapy, it remains to be determined whether BM hemorrhage is a therapeutic response and may affect patients’ survival or not [16,17].

The aims of the study were twofold: (1) to explore any risk factors associated with hemorrhage in BMs from lung adenocarcinomas and (2) to determine whether BM hemorrhage can predict patients’ overall survival.

## 2. Materials and Methods

### 2.1. Participants

**Figure 1:**
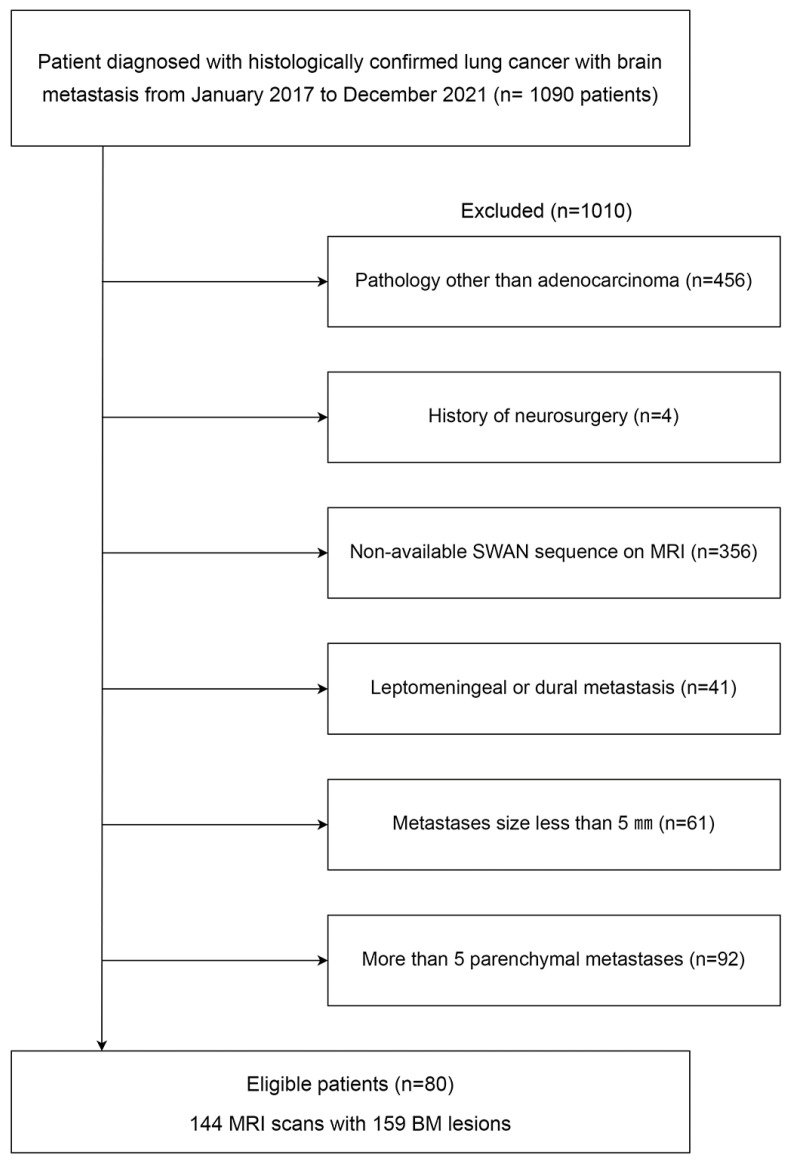
Flow chart of patient enrollment. BM: brain metastasis; MRI: magnetic resonance imaging; SWAN: susceptibility-weighted angiography.

This retrospective study was approved by the institutional review board, which waived the requirement for informed patient consent (3-2021-0418). We retrospectively searched electronic medical records to identify magnetic resonance imaging (MRI) sets of patients with histologically confirmed lung cancer and BM from January 2017 to May 2022. After identifying 1090 potential participants, the following exclusion criteria were applied: (1) histologic subtypes of primary lung cancer other than adenocarcinoma (*n* = 456), (2) history of neurosurgery (*n* = 4), (3) lack of susceptibility-weighted angiography (SWAN) MRI of the brain for evaluation of the hemorrhage (*n* = 356), (4) leptomeningeal or dural metastasis (*n* = 41), (5) absence of “evaluable BM” for the hemorrhage (*n* = 61), and (6) more than five BMs in a patient, as clustered data may lead a statistical bias (*n* = 92). “Evaluable BM” was defined as BM where the longest diameter was >5 mm. After the exclusion criteria were applied, a total of 80 patients with 144 MRI scans and 159 BMs were included in this study (Figure 1).

Histopathological diagnoses of lung cancer were obtained using bronchoscopic, percutaneous needle-guided, or surgical biopsies for all patients. To determine *EGFR* mutation status, DNA was extracted from formalin-fixed paraffin-embedded tissues using a DNeasy isolation kit (Qiagen, Valencia, CA, USA), according to the manufacturer’s instructions. For *EGFR*, direct DNA sequencing of exons 18–21 was performed, or the PNAClamp^TM^ EGFR Mutation Detection Kit (PANAGENE, Daejeon, Korea) was used. Each case was classified as positive or negative for a mutation by comparing with the wild-type sequence. Anaplastic lymphoma kinase (*ALK*) fusion was identified using the Ventana ALK (D5F3) CDx Assay. ROS1 fusion was screened using immunohistochemistry staining and confirmed using an AmoyDx ROS1 Gene Fusion Detection Kit [18]. All data were anonymized, and all experiments were conducted in accordance with approved guidelines.

NSCLC staging was performed in accordance with the 8th edition of the American Joint Committee on Cancer guidelines [19]. Tumor–node–metastasis (TNM) staging of lung cancer at diagnosis was based on computed tomography (CT) scans of the chest and abdomen, whole-bone scanning, and positron emission tomography–CT, which were performed during the initial evaluation of NSCLC.

### 2.2. MRI and Image Analysis

**Figure 2:**
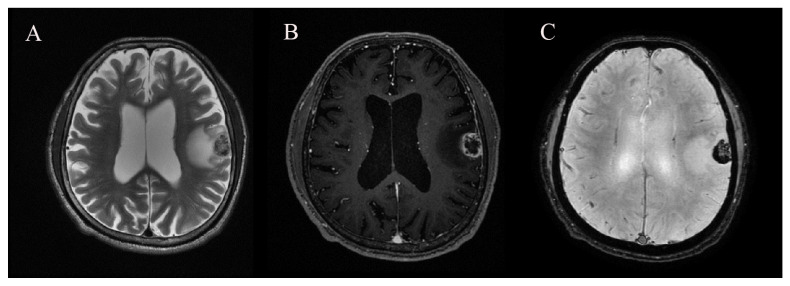
A 67-year-old man with hemorrhagic brain metastasis (BM) in left frontoparietal lobe. (**A**) T2-weighted imaging, (**B**) contrast-enhanced 3D T1-weighted imaging, and (**C**) susceptibility-weighted angiography (SWAN).

Routine MRI scans for evaluating BMs were performed using a GE 3T Discovery MR750 (GE Healthcare, Milwaukee, WI, USA) scanner. The brain MRI protocol used in this study included three-dimensional (3D) T1-weighted imaging, T2-weighted imaging, fluid-attenuated inversion recovery, SWAN, and subsequent contrast-enhanced 3D T1-weighted imaging. The sequence parameters for SWAN were as follows: repetition time (TR) = 31 ms, echo time (TE) = 3 echoes centered around 23 ms, flip angle = 10°, slice thickness = 2 mm, intersection gap = 0 mm, field of view = 210 mm, matrix number = 320 × 224, and bandwidth = 62.50 kHz (Figure 2).

Two radiologists, who were blinded to the clinical and histopathologic findings, independently evaluated the MR images on the picture archiving and communication system workstation monitors for the following characteristics: number of metastatic lesions, size of the metastatic lesions, and presence of intratumoral hemorrhage. The lesion size was defined as the lesion’s largest dimension in any plane on the MR image. Intratumoral hemorrhage was considered to be present if the lesion contained dark signals on the phase map of SWAN [20]. All disagreements were resolved by consensus.

### 2.3. Statistical Analyses

The cohort was divided into two groups: non-hemorrhagic and hemorrhagic BMs. Each patient’s age, sex, smoking history (never smoker vs. ever smoker), hypertension, antithrombogenic agents, time interval to BM, TNM classification, cancer stage, molecular subtype (EGFR, ALK, and ROS1), treatment type (including radiotherapy and target agents), and lesion size were compared between both groups using a generalized estimating equation (GEE). The advantage of using GEE is that it accounts for possible dependence among clinical variables and MRI measures within the patients who tend to be alike [21]. Multivariate GEE analysis was also performed to adjust for age, sex, tumor size, *EGFR* mutation, and treatment options, which were statistically significant in the univariate analysis or were basic clinical factors. To determine the influence of variables in predicting hemorrhage within BMs, variable importance scores were calculated using a gradient boosting algorithm (GBM).

To explore the predictability of BM hemorrhage for overall survival after BM (OSBM), Kaplan–Meier analysis was performed. Log-rank tests were used to compare non-hemorrhagic and hemorrhagic BMs. For subgroup analysis, we split our cohort into patients with single BM and multiple BMs. Then, survival analyses were also performed according to presence of intracranial hemorrhage in each subgroup. Interrater reliability was assessed using the intraclass correlation coefficient with a two-way random model of absolute agreement. Statistical significance was set at *p*-value < 0.05. All data analyses were performed using R version 3.5.3.

## 3. Results

### 3.1. Hemorrhagic Versus Non-Hemorrhagic BMs from Lung Adenocarcinoma

Clinical characteristics were compared between the two groups (Table 1). The mean size of hemorrhagic BMs was significantly larger than that of non-hemorrhagic BMs (16.1 ± 11.7 vs. 11.7 ± 9.7 mm, *p* = 0.03). Radiation or TKI therapy was marginally associated with hemorrhage in BMs (*p* = 0.06). Moreover, patients with hemorrhagic BMs were more likely to have received a combination of TKI and radiation therapy than those with non-hemorrhagic BMs (40.9% vs. 19.0%, *p* = 0.03). No significant differences were observed in the type of TKI, brain radiotherapy, and immunotherapy; TNM staging of lung cancer at the initial diagnosis; time interval from the initial diagnosis of lung cancer to BM surveillance; *EGFR*, *ALK*, and *ROS1* mutations; age; hypertension; antithrombogenic agent; and sex of patients.

### 3.2. Most Influential Variable Predicting BM Hemorrhage

Multivariate GEE analysis found that hemorrhage in BMs was independently associated with combined TKI and radiation therapies (odds ratio (OR) = 2.31, 95% confidence interval (CI): 1.13, 4.71, *p* = 0.02) and BM size (OR = 1.07, 95% CI: 1, 1.13, *p* = 0.03, Table 2). In the GBM, BM size demonstrated the highest predictive power for hemorrhage in BM, followed by age, combination therapy, TKI therapy, radiation therapy, sex, time interval, and *EGFR* mutation (Table 3).

### 3.3. Effect of BM Hemorrhage on Overall Survival after BM of Patients

**Figure 3:**
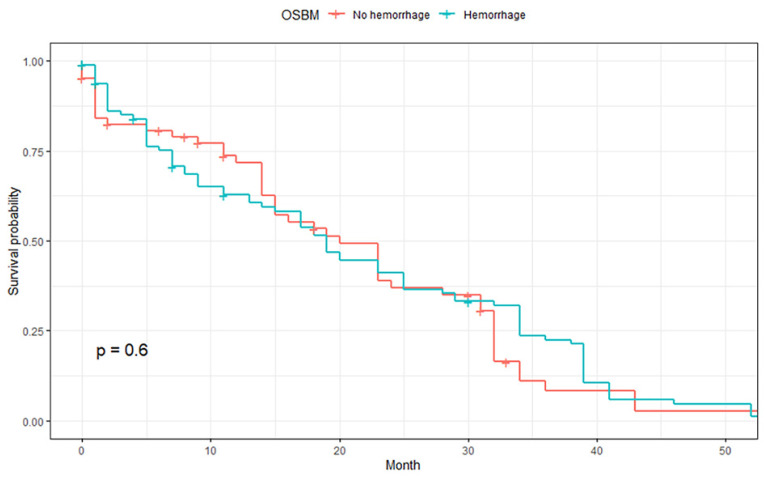
Comparison of overall survival after brain metastasis (OSBM) between hemorrhagic and non-hemorrhagic BMs.

The median OSBM was 23.0 months, and no significant difference in OSBM was observed between those with non-hemorrhagic and hemorrhagic BMs (*p* = 0.6, Figure 3). In subgroup analyses, there was no significant difference in OSBM depending on intracranial hemorrhage in patients with single BM (*p* = 0.42) as well as multiple BMs (*p* = 0.8, Supplemental Figure S1).

## 4. Discussion

In this study, we explored the risk factors for spontaneous hemorrhage in BM from lung adenocarcinoma and assessed whether hemorrhage in BM may affect patient survival. The results indicated that hemorrhage was more likely to occur in larger BMs, and a combination of TKI and radiation therapies increased the risk of hemorrhage in BMs. However, hemorrhage in BMs was not significantly associated with OSBM. These results may contribute to elucidating the pathophysiology of hemorrhage in BMs from lung adenocarcinomas. These results may also have a clinical impact as neurologic deterioration could be prevented by identifying patients with a high risk of developing BM hemorrhage.

The pathophysiology of hemorrhage in BMs remains unclear. One theory is that abnormal tumor vascularization may play an important role as newly formed vessels within the tumor mass are characterized by numerous structural abnormalities and may be thin-walled, poorly formed, or dilated, leading to their dysfunction [22]. In metastatic brain tumors, vascular endothelial growth factor (VEGF) and metalloproteinase 2-mediated hypoxic signaling pathway may result in the loss of vascular integrity, leading to tumor-associated hemorrhage and necrosis [23,24]. The results of a previous study are similar to our finding that bleeding occurrence may increase with tumor size [25]. We infer that after increasing to a certain size, the tumor experiences hypoxia, driving the production of angiogenic factors such as VEGF. Therefore, larger BMs may be more vulnerable to hemorrhage [26].

There have been contradictory findings on the relationship between radiation therapy and the risk of hemorrhage. A study identified hemorrhage in 7.4% of BMs before radiosurgery and in 18.5% of BMs after radiosurgery, suggesting that stereotactic radiosurgery (SRS) may increase the risk of hemorrhage in BMs [27]. Moreover, intratumor hemorrhage has been reported after radiosurgery in patients with BMs [28,29]. In contrast, other studies have shown that radiosurgery is not significantly related with hemorrhage in BMs [30,31]. Furthermore, whole-brain radiation therapy (WBRT) alone has been found to decrease hemorrhagic events [32,33]. The results of our study suggest that patients undergoing prior radiation therapy were more likely to have a hemorrhage in BMs than patients who did not undergo prior radiation therapy, although this result was not statistically significant. The tendency for hemorrhage after radiation therapy could be due to endothelial cell damage caused by the irradiation, resulting in the release of various cytokines, which may produce thrombotic lesions or disrupt the blood–brain barrier, leading to hemorrhage [34]. Additionally, an increase in the intravascular outflow resistance, resulting from radiation-induced venous obliteration, could promote the occurrence of hemorrhage [35].

We also found that EGFR-TKI therapy was associated with hemorrhage in BMs from lung adenocarcinomas, particularly when combined with radiation therapy. In a case report of two patients with lung cancer, hemorrhagic BMs developed 1 month after a combination of EGFR-TKI and radiation therapies [36]. Thus, the combination of EGFR-TKI and radiation therapies may be the cause of hemorrhage in BMs. Increasing evidence has demonstrated that EGFR-TKIs are a radiation sensitizer in the treatment of NSCLC, head and neck, breast, and colorectal cancers [37,38]. We believe that EGFR-TKI may reinforce the role of radiation-induced endothelial injury and subsequent hemorrhage. In addition, although statistically not significant, more patients with hemorrhagic BMs received 3rd generation TKI compared with those with non-hemorrhagic BMs (24.5% vs. 0.0%, *p* = 0.14, Supplemental Table S1). This finding is noticeable as 3rd generation TKI has a better brain penetrance than 1st and 2nd generation TKIs [39]. However, this hypothesis requires further validation in future studies with larger population.

Bleeding risk varies depending on histologic diagnosis. Hemorrhage is most commonly observed in melanoma BM, with the hemorrhage rate ranging from 9% to 30% [30,40]. BMs from renal carcinoma, choriocarcinoma, and papillary carcinoma of the thyroid also frequently exhibit intratumoral hemorrhage [11,13,41]. A few studies have reported on hemorrhage in lung cancer BMs. A previous study reported that the incidence of hemorrhage in NSCLC BM is low (approximately 1.2%); however, the true incidence of hemorrhage is likely higher than that reported [42]. Our study demonstrated that the incidence of hemorrhage was 60.1%. There are a number of explanations for the large discrepancy between the two studies. First, this study used the SWAN sequence for the detection of hemorrhage, which is sensitive to bleeding. The results of another study using the susceptibility-weighted imaging sequence also support these findings; 68.9% of patients with lung cancer BM showed intratumoral hemorrhage [43]. Second, the size of BM used in this study was larger than that of the previous study as we excluded small BMs (with the longest diameter less than 5 mm). Third, it is unlikely that the previous study accounted for the effect of a combination of EGFR-TKI and radiation therapies because EGFR-TKIs have been recently implemented.

Hypertension, current smoking and antithrombogenic medication are significant risk factors for intracranial hemorrhage [14,15,44,45,46]. However, our study demonstrated that these clinical factors are not associated with intratumor hemorrhage of BM. Previous studies also reported that there is no additional risk for intracranial hemorrhage attributed to the use of anticoagulation in patients with BMs [47,48]. We may speculate that this phenomenon can be explained by different vasculature of BMs from that of normal tissue as well as primary lung cancer [49].

Intratumor hemorrhage could be an after effect of rapid tumor growth, but it could also be a response to therapy. The effect of BM hemorrhage on patient prognosis is clinically important. However, according to the results of this study, there was no significant difference in OSBM between the hemorrhagic and non-hemorrhagic groups.

This study had limitations. First, it was a retrospective case-control study and might not be sufficient to relate BM hemorrhage to particular exposures. A prospective longitudinal study is required to support our results. Second, our results apply to “Evaluable BM” with the longest diameter >5 mm. Thus, the relationship between hemorrhage and small BMs is still unresolved. Third, to avoid bias from clustered data, we used generalized estimating equation and excluded patients with more than five BMs. Thus, our results might not be applied in patients with more than five BMs. Fourth, although our results indicated that hemorrhage was not associated with OSBM, this should be carefully interpreted because we did not exclude hemorrhage as the cause of death with post-mortem analysis. Finally, the results were obtained from a single center and may have limited generalizability.

## 5. Conclusions

We demonstrated that hemorrhage in BMs from NSCLC adenocarcinomas may be associated with larger BM size and combination therapy involving intracranial radiation exposure and TKI use. However, hemorrhage was not associated with patient prognosis. These findings may help explain the mechanism by which hemorrhage occurs in lung adenocarcinoma BM and aid clinicians in preventing BM hemorrhage or its aggravation. Particularly, a clinician is required to closely monitor patient with large BMs of if they have received combination therapy of EGFR-TKI and radiation therapy. However, stopping the antithrombotic agent may not be necessary to prevent BM hemorrhage.

## Figures and Tables

**Table 1:** Clinical characteristics of patients with brain metastases (BMs) from lung adenocarcinoma.

	Non-Hemorrhagic BMs (*n*= 63)	Hemorrhagic BMs (*n* = 96)	Total (*n* = 159)	*p*
Age (years)	67.7 ± 14.7	66.3 ± 12.2	66.8 ± 13.2	0.5
Sex				0.75
Female	35 (55.6%)	44 (45.8%)	79 (49.7%)	
Male	28 (44.4%)	52 (54.2%)	80 (50.3%)	
Smoking				0.93
Never smoker	44 (69.8%)	69 (71.9%)	113 (71.1%)	
Ever smoker	19 (30.2%)	27 (28.1%)	46 (28.9%)	
Hypertension				0.46
No	39 (61.9%)	60 (62.5%)	99 (62.3%)	
Yes	24 (38.1%)	36 (37.5%)	60 (37.7%)	
Antithrombotic treatment				0.2
No	50 (79.4%)	86 (89.6%)	136 (85.5%)	
Yes	13 (20.6%)	10 (10.4%)	23 (14.5%)	
Interval from initial diagnosis of lung cancer to BM surveillance (months)	13.1 ± 14.7	19.0 ± 25.1	16.7 ± 21.8	0.09
BM size (mm)	11.7 ± 9.7	16.1 ± 11.7	14.4 ± 11.1	0.03 *
TNM stage				0.13
Stage 1	8 (12.7%)	5 (5.3%)	13 (8.2%)	
Stage 2	3 (4.8%)	4 (4.2%)	7 (4.4%)	
Stage 3	15 (23.8%)	13 (13.7%)	28 (17.7%)	
Stage 4	37 (58.7%)	73 (76.8%)	110 (69.6%)	
EGFR				0.25
Negative	27 (45.8%)	29 (31.9%)	56 (37.3%)	
Positive	32 (54.2%)	62 (68.1%)	94 (62.7%)	
ALK				0.72
Negative	48 (92.3%)	66 (95.7%)	114 (94.2%)	
Positive	4 (7.7%)	3 (4.3%)	7 (5.8%)	
ROS1				1
Negative	60 (100.0%)	87 (98.9%)	147 (99.3%)	
Positive	0 (0.0%)	1 (1.1%)	1 (0.7%)	
TKI therapy				0.06
No	44 (69.8%)	43 (44.8%)	87 (54.7%)	
Yes	19 (30.2%)	53 (55.2%)	72 (45.3%)	
TKI subtype				0.08
1st generation	9 (47.4%)	18 (34.0%)	27 (37.5%)	
2nd generation	10 (52.6%)	22 (41.5%)	32 (44.4%)	
3rd generation	0 (0.0%)	13 (24.5%)	13 (18.1%)	
Radiation therapy for BM				0.06
No	34 (54.0%)	35 (36.5%)	69 (43.4%)	
Yes	29 (46.0%)	61 (63.5%)	90 (56.6%)	
Subtype of radiation therapy				0.26
SRS	5 (17.2%)	16 (26.2%)	21 (23.3%)	
WBRT	24 (82.8%)	45 (73.8%)	69 (76.7%)	
Immunotherapy				0.34
No	48 (96.0%)	71 (92.2%)	119 (93.7%)	
Yes	2 (4.0%)	6 (7.8%)	8 (6.3%)	
Combination therapy				0.03 *
Neither TKI nor radiation therapy	27 (42.9%)	21 (21.9%)	48 (30.2%)	
TKI or radiation therapy	24 (38.1%)	36 (37.5%)	60 (37.7%)	
Both TKI and radiation therapy	12 (19.0%)	39 (40.6%)	51 (32.1%)	

BM: brain metastasis; TNM: tumor–node–metastasis; EGFR: epidermal growth factor receptor; ALK: anaplastic lymphoma kinase; TKI: tyrosine kinase inhibitor; SRS: stereotactic radiosurgery; and WBRT: whole-brain radiation therapy. Asterisk (*) indicates a *p*-value < 0.05.

**Table 2:** Multivariate generalized estimating equation (GEE) analysis for hemorrhage occurrence in brain metastases (BMs).

	OR (95% CI)	*p*-Value
BM size (mm)	1.07 (1–1.13)	0.03 *
Combination therapy	2.31 (1.13–4.71)	0.02 *
Age	0.99 (0.96–1.01)	0.26
Sex	0.86 (0.3–2.48)	0.78
EGFR	1.22 (0.4–3.76)	0.72

OR: odds ratio; CI: confidence interval; BM: brain metastasis; and EGFR: epidermal growth factor receptor. Asterisk (*) indicates a *p*-value < 0.05.

**Table 3:** Relative influence of variables for prediction of hemorrhagic brain metastases (BMs) using gradient boosted model (GBM).

Variables	Relative Influence
BM size	49.83
Age	16.65
Combination therapy	13.81
TKI therapy	10.00
Radiation therapy	5.35
Sex	2.88
Time interval	1.44
EGFR	0

BM: brain metastases; TKI: tyrosine kinase inhibitor; and EGFR: epidermal growth factor receptor.

## Data Availability

The data can be shared up on request.

## Supplementary Materials

The following supporting information can be downloaded at: https://www.mdpi.com/article/10.3390/cancers15030619/s1, Table S1: Comparison of TKI subtypes between non-hemorrhagic and hemorrhagic BMs; Figure S1: Overall survival after brain metastasis (OSBM) depending on intracranial hemorrhage in patients with single BM (A) and multiple BMs (B).
