# Supplementary material for: Associated Factors of Spontaneous Hemorrhage in Brain Metastases in Patients with Lung Adenocarcinoma
Source: Cancers (Basel). 2023 Jan 19;15(3):619. doi: 10.3390/cancers15030619 (PMC9913139; doi:10.3390/cancers15030619)

Table S1. Comparison of TKI subtypes between non-hemorrhagic and hemorrhagic BMs.

	Non-hemorrhagic BMs (N=19)	Hemorrhagic BMs (N=40)	Total (N=39)	p
TKI subtype				0.66
1st generation	9 (47.4%)	18 (45.0%)	27 (45.8%)	
2nd generation	10 (52.6%)	22 (55.0%)	32 (54.2%)	
	Non-hemorrhagic BMs (N=19)	Hemorrhagic BMs (N=53)	Total (N=72)	p
TKI subtype				0.14
1st and 2nd generation	19 (100.0%)	40 (75.5%)	59 (81.9%)	
3rd generation	0 (0.0%)	13 (24.5%)	13 (18.1%)	

Figure S1. Overall survival after brain metastasis (OSBM) depending on intracranial hemorrhage in patients with single BM (A) and multiple BMs (B).

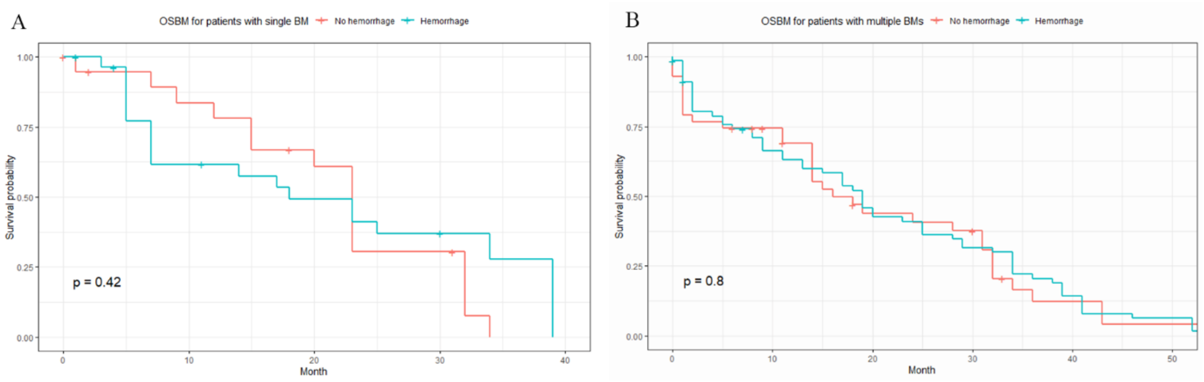

Supplement: Supplementary file 1 [file cancers-15-00619-s001.zip › cancers-2160185-supplementary.pdf]
